# Identification of quantitative trait nucleotides and candidate genes for soybean seed weight by multiple models of genome-wide association study

**DOI:** 10.1186/s12870-020-02604-z

**Published:** 2020-09-01

**Authors:** Benjamin Karikari, Zili Wang, Yilan Zhou, Wenliang Yan, Jianying Feng, Tuanjie Zhao

**Affiliations:** National Center for Soybean Improvement, Key Laboratory of Biology and Genetic Improvement of Soybean (Ministry of Agriculture), State Key Laboratory of Crop Genetics and Germplasm Enhancement, Nanjing Agricultural University, Nanjing, 210095 People’s Republic of China

**Keywords:** Association mapping, Complex nature, Haplotype, Hub genes, Co-expression network

## Abstract

**Background:**

Seed weight is a complex yield-related trait with a lot of quantitative trait loci (QTL) reported through linkage mapping studies. Integration of QTL from linkage mapping into breeding program is challenging due to numerous limitations, therefore, Genome-wide association study (GWAS) provides more precise location of QTL due to higher resolution and diverse genetic diversity in un-related individuals.

**Results:**

The present study utilized 573 breeding lines population with 61,166 single nucleotide polymorphisms (SNPs) to identify quantitative trait nucleotides (QTNs) and candidate genes for seed weight in Chinese summer-sowing soybean. GWAS was conducted with two single-locus models (SLMs) and six multi-locus models (MLMs). Thirty-nine SNPs were detected by the two SLMs while 209 SNPs were detected by the six MLMs. In all, two hundred and thirty-one QTNs were found to be associated with seed weight in YHSBLP with various effects. Out of these, seventy SNPs were concurrently detected by both SLMs and MLMs on 8 chromosomes. Ninety-four QTNs co-localized with previously reported QTL/QTN by linkage/association mapping studies. A total of 36 candidate genes were predicted. Out of these candidate genes, four hub genes (*Glyma06g44510, Glyma08g06420, Glyma12g33280* and *Glyma19g28070*) were identified by the integration of co-expression network. Among them, three were relatively expressed higher in the high HSW genotypes at R5 stage compared with low HSW genotypes except *Glyma12g33280*. Our results show that using more models especially MLMs are effective to find important QTNs, and the identified HSW QTNs/genes could be utilized in molecular breeding work for soybean seed weight and yield.

**Conclusion:**

Application of two single-locus plus six multi-locus models of GWAS identified 231 QTNs. Four hub genes (*Glyma06g44510****,*** *Glyma08g06420****,*** *Glyma12g33280* & *Glyma19g28070*) detected via integration of co-expression network among the predicted candidate genes.

## Background

The world’s human population is estimated to reach 10 million by 30-year time [1] with increasing abiotic and biotic stress as well as reduction in arable land for agricultural activities [2]. This implies that food and nutritional security is under threat. Legume crops including soybean (*Glycine max* L. Merr.) play a significant role in ensuring global food and nutritional security in addition to their abilities to improve soil quality through nitrogen fixation [3]. Consumption of legume crops is associated with health and physiological benefits like prevention of cardiovascular diseases, obesity, diabetes mellitus, cancer and relief of menopausal symptoms [4–6]. However, the insufficient soybean production in China and many under-developing countries is a big challenge and per unit yield of soybean needs to be improved rapidly.

One of the major determinants of soybean yield, seed use and evolutionary fitness is seed weight [7–9]. For example, large-seeded cultivars are used for boiled soybean (*nimame*), green soybean (*edamame*), soymilk and soybean curd (*tofu*), while small-seeded cultivars are suitable for fermented soybean (*nattō*) and sprout production [10–12]. Soybean breeders need to create big variation of seed weight for selection of varieties with different end-use purposes. Seed weight is also an important trait that was targeted during soybean domestication [13–16], and the range of 100-seed weight can vary from less than 1 *g* in wild soybean (*Glycine soja* Sieb. et Zucc.) accessions to more than 60 *g* in some specific *edamame* varieties.

As a complex quantitative trait, seed weight is assumed to be controlled by several major genes/loci plus numerous undetectable loci with minor effects (thus, polygenes), interacting with environments. More than 300 quantitative trait loci /nucleotides (QTL/QTNs) for soybean seed weight have been reported on SoyBase (www.soybase.org) via linkage mapping. However, integrating results from linkage mapping into breeding program is challenging due to the higher confidence interval and less genetic variation [17]. As a result in the recent years, marker-trait association is used to take advantage of all recombination events that occur in the evolutionary history of a natural population based on linkage disequilibrium (LD) [18, 19]. Marker-trait association allows researchers to utilize natural diversity and locate valuable genes in the genome [18]. For instance, Miao et al. [20] recently applied regional association mapping for seed oil and identified *GmSWEET39* (*Glyma.15 g049200/Glyma15g05470)* which was subsequently overexpressed in Arabidopsis leading to at least 10% increase in seed oil content.

Tens of QTNs have been detected and reported through genome-wide association studies (GWAS) across the 20 chromosomes [13, 21–30]. However, different mapping results can be obtained due to population type, size and GWAS method. Single-marker genome-wide scan models such as mixed linear model (MLM) and general linear model (GLM) are mostly frequently used in genetic dissection of soybean seed weight. These models have certain limitations including the issue of multiple test correction for threshold value of significance, and mapping power [31]. A number of multi-locus models have been developed and applied in recent GWAS in several crops including soybean. Six of such models (mrMLM, FASTmrMLM, FASTmrEMMA, pLARmEB, pKWmEB & ISIS EM-BLASSO) are implemented in R with the mrMLM.GUI package [32]. These models have become the state-of-the-art procedure to identify QTNs with complex traits due to their detection power and robustness [33–37].

Bioinformatics tools have enhanced easy identification of potential genes for target QTL. One of the strategies is to utilize co-expression network which aims at prioritizing functionally related genes. It has been successfully used in several crops such as maize [38], rice [39], peanut [40], Arabidopsis [41], soybean [42, 43], among others. By integration of co-expression network analysis, a class of hub genes which induce major transcriptome reprogramming during grapevine development were identified Palumbo et al. [44]. The hub genes (genes highly connected) may give clue on the role of those genes in the network [45].

In most of the earlier reported GWAS for seed weight, population sizes were mostly < 500 [29, 30, 46–48]. The population size, genetic diversity as well as genome coverage/number of SNPs, linkage disequilibrium, and statistical methods used have been reported to affect the power of GWAS [17, 49, 50]. Therefore, our present study utilized 573 breeding lines with 61,166 SNPs to conduct marker-trait association viz., two single-locus models (SLMs) and six multi-locus models (MLMs) to identify significant SNPs. Also, potential candidate genes were predicted, out of which hub genes were identified by the integration of functional co-expression network. Application of multiple models of GWAS detected 231 QTNs, out of which 94 co-localized with earlier reported QTL/QTNs. This demonstrate the use of multiple models of GWAS to unravel the complex architecture of seed weight in our recently developed diverse breeding lines.

## Results

### Phenotypic variation of HSW in the YHSBLP

**Fig. 1:**
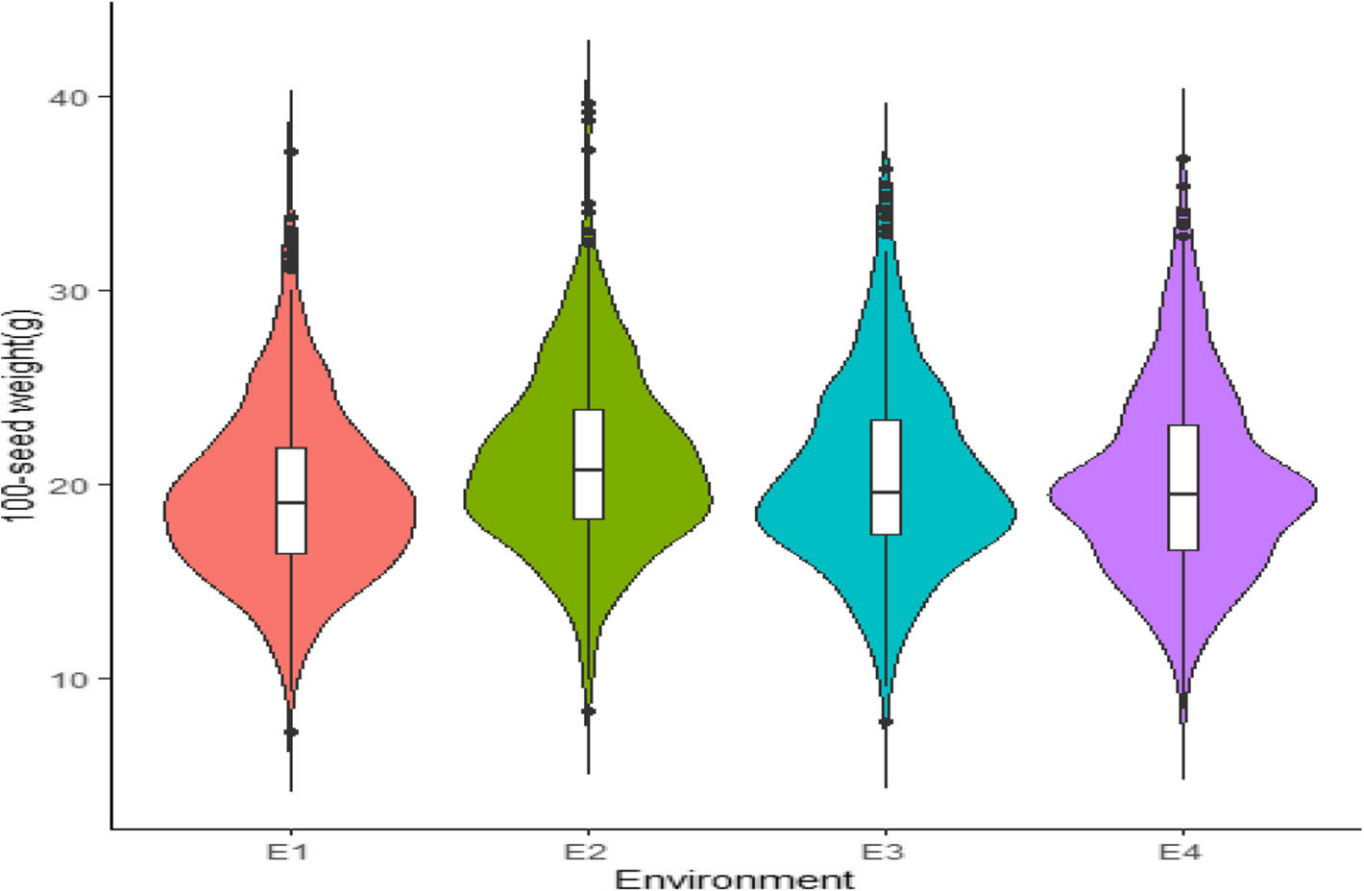
Variation of HSW in each of the environments. The black in the middle of the box shows the median, the white box indicates the range from the lower quartile to the upper quartile, the black line represents the dispersion and frequency distribution of the phenotypic data. The black dots represent phenotypic data that were extreme in each environment

The phenotypic variation of HSW in YHSBLP across the four environments (E1, E2, E3 and E4) followed a normal distribution, typical of quantitative traits (Fig. 1). In the E1, HSW ranged from 7.24–37.19 g with the mean of 19.40 ± 4.47 g whereas E2, E3 and E4 had a range (mean ± standard deviation) of 8.23–39.70 g (21.22 ± 4.62 g), 7.71–36.32 g (20.43 ± 4.80 g) and 8.38–36.78 g (20.09 ± 4.78 g), respectively (Additional File 1: Table S1). The HSW was significantly (*P* < 0.001) affected by genotype, environment and genotype by environment interaction (Table 1). The broad-sense heritability (*h*^2^) was 98.53%. These suggest that HSW in the summer sowing of YHSBLP was mainly influenced by genetic factors with less effect by environmental factors (Table 1).

**Table 1:** Joint ANOVA for HSW across the 4 environments

Source	*DF*	*SS*	*MS*	*F* Value	*P* value	*h*^*2*^
Environment	3	15,355.00	5118.39	5.26	0.0261	98.53%
Rep(Environment)	8	7871.95	983.99	715.38	< 0.0001	
Genotype	572	119,437.00	208.81	17.64	< 0.0001	
Genotype×Environment	1665	19,977.00	12.00	8.72	< 0.0001	
Residual	4228	5849.90	1.38			

### SNPs distribution and population structure in the YHSBLP

**Fig. 2:**
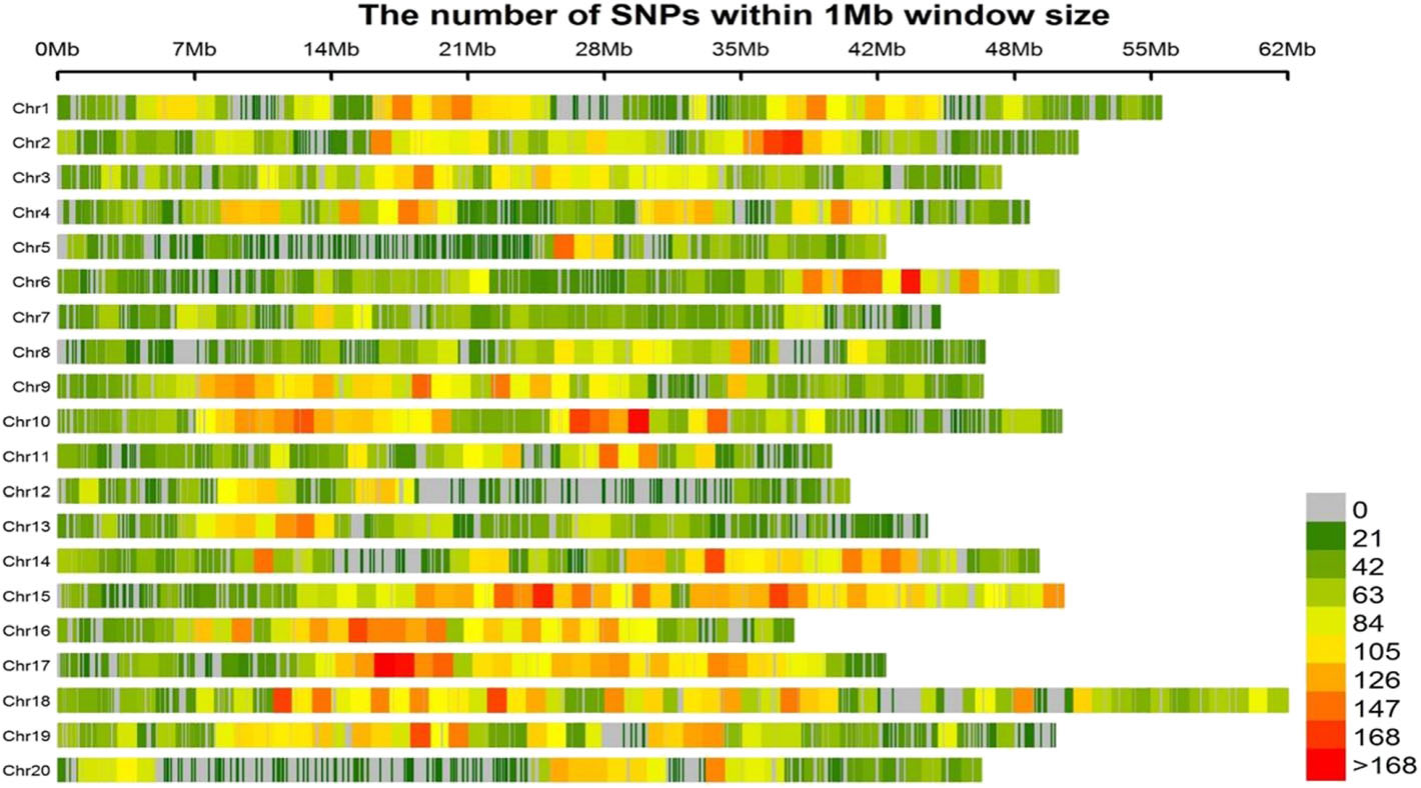
Distribution of 61,166 SNPs on the 20 chromosomes of soybean. The horizontal axis shows chromosome length (Mb); the vertical axis gives the chromosome number and the different colors depict SNP density (the number of SNPs per window)

All the 573 accessions were genotyped by RAD-seq technology. After removing monomorphic markers as well as markers with MAF < 5% and missing and heterozygous allele rate > 30%, a total of 61,166 SNPs were used for this study. The total length of the genome was 950,068,807 bp (950.07 Mb) representing 85.21% of the genome of soybean. The range of the number of SNPs per chromosome was 1467–4844 with chromosome 5 and 18 having the least and highest, respectively. The highest and lowest SNP density of 91.9 SNPs/Mb and 35 SNPs/Mb were found on chromosome (Chr.) 15 and Chr.05, respectively, with the longest chromosome being Chr.18 (Fig. 2).

**Fig. 3:**
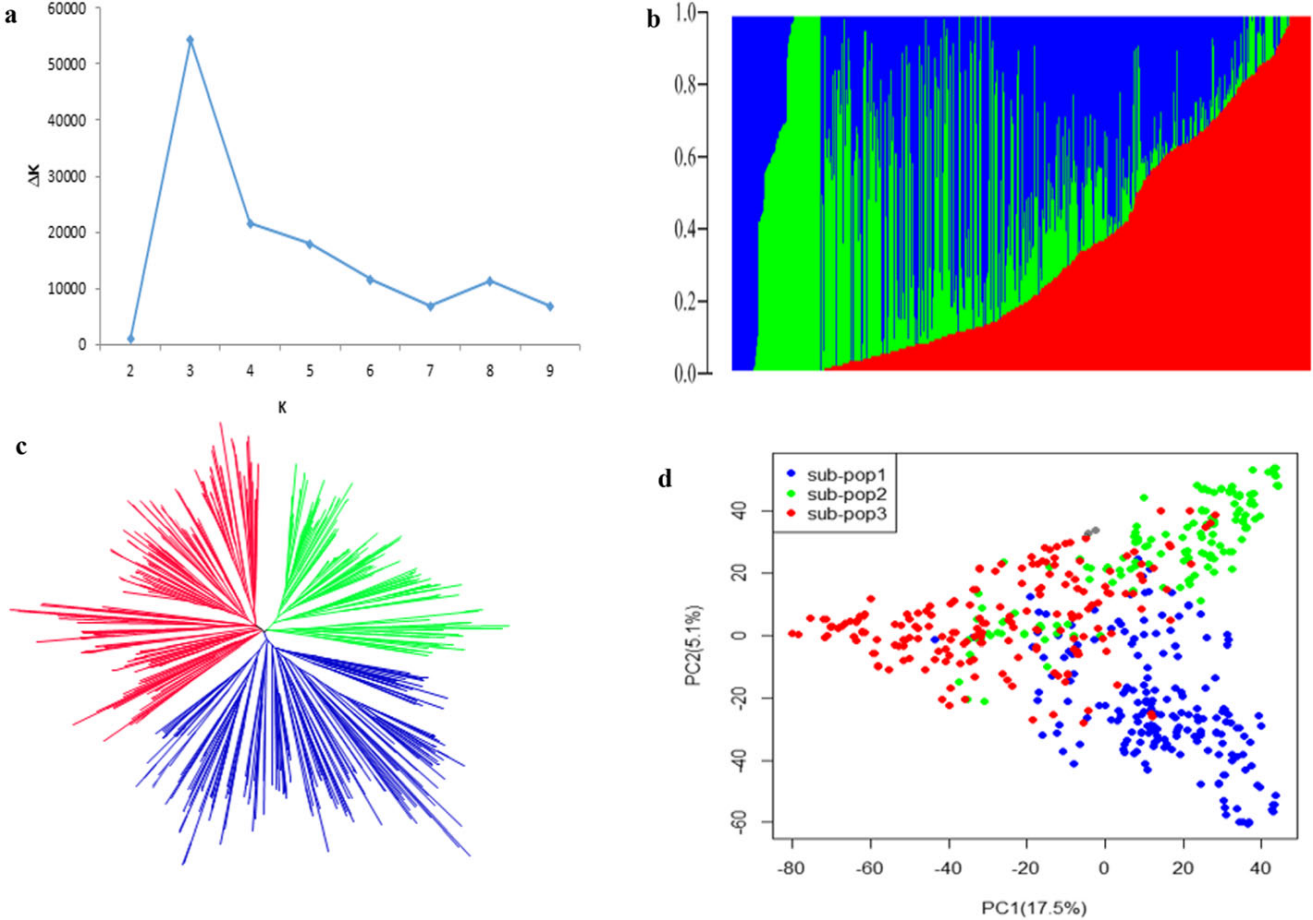
Population structure of 573 accessions. **a** Plot of ΔK calculated for K = 1–10. **b** Population structure obtained from ADMIXTURE software. Three colors (blue, green and red) represent three subpopulations. Each color represents one inferred ancestral population. Each vertical column represents one individual and colored segment in each column represents percentage of the individual inferred ancestral population in the YHSBLP. **c** A neighbor-joining tree of the YHSBLP with three clusters. **d** Principal Component Analysis plot

The 573 accessions were grouped into three subpopulations as evident by population structure obtained from ADMIXTURE software (Fig. 3a & b), phylogenetic analysis (Fig. 3c) and PCA (Fig. 3d). The first two PC accounted for 22.60% variation (Fig. 3d). The lines with the probability (Q) more than 0.70 score were considered as pure lines while those with Q ≤ 0.7 were considered as admixtures. The subpopulation 1 comprised 107 pure lines with an average HSW of 17.68 g. The subpopulation 2 and 3 consisted of 101 and 92 pure lines, respectively, with average HSW of 22.95 g and 19.73 g in that same order. The HSW differed significantly among the subpopulations. The remaining 273 admixtures had average HSW of 20.52 g.

### SNP-trait association mapping

**Fig. 4:**
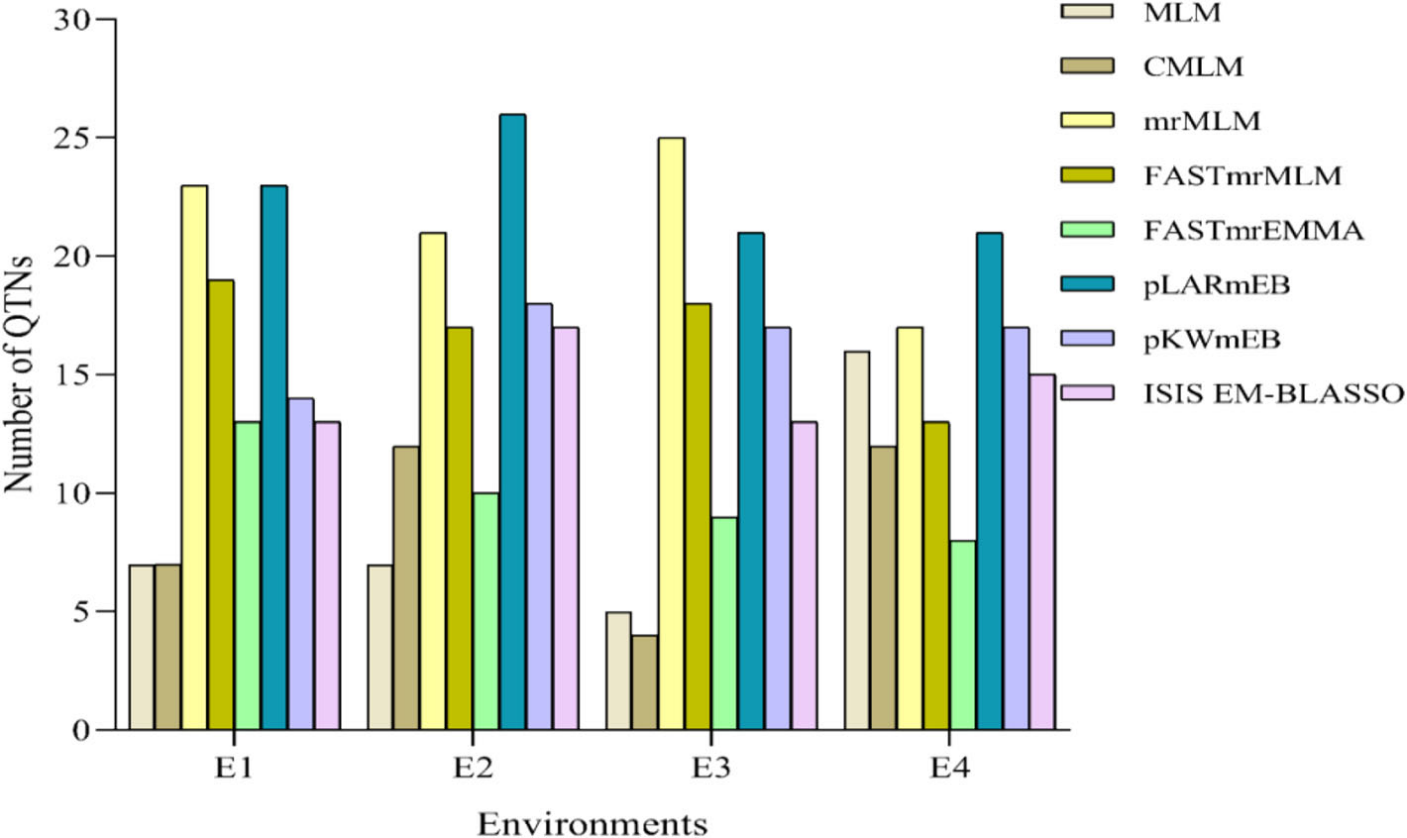
Total number of SNPs detected by the 8 models in each environment. The X and Y-axis represent the environments (E1, E2, E3 & E4) and number of SNPs detected, respectively

A total of 39 SNPs significantly associated with HSW in at least one environment were detected via MLM (K + Q) and CMLM (K + PCA), respectively, with −*log*_10_(*P*) =4.00–12.25 (Additional File 2: Table S2; Additional File 3: Fig. S1; Additional File 4: Fig. S2). Among the 39 loci, twenty-four were detected by both models (MLM and CMLM) (Additional File 2: Table S2; Additional File 5: Fig. S3A). These QTNs were distributed unevenly on 11 chromosomes. Each of the models (MLM and CMLM) detected the highest number of six QTNs on Chr.02 and Chr.13, respectively. Out of these QTNs, seven were detected in two environments by the two SLMs on five chromosomes. Chromosome 14 harbored three QTNs (*qHSW-14-5, qHSW-14-10* & *qHSW-14-11*) whereas one QTN each was harbored on Chr08 (*qHSW-8-8*), Chr.13 (*qHSW-13-26*), Chr.15 (*qHSW-15-4*) and Chr.16 (*qHSW-16–5*) (Additional File 2: Table S2). The highest number of 16 QTNs were detected in environment (E4) by MLM whereas CMLM detected 12 QTNs in either E2 or E4 (Fig. 4). Most of the SNPs detected overlapped with QTL detected by earlier linkage mapping studies published on SoyBase and some association mapping studies (Additional File 2: Table S2).

Six MLMs viz. mrMLM, FASTmrMLM, FASTEMMA, pLARmEB, pKWmEB and ISIS EM-BLASSO detected a total of 209 QTNs across all the 20 chromosomes unevenly with the range of logarithm of odd (*LOD*) = 3.01–17.31 (Additional File 2: Table S2). The highest number of 13 QTNs were detected on Chr.13 by mrMLM whereas 25 QTNs were identified in E2 by pLARmEB (Fig. 4; Additional File 2: Table S2). The number of QTNs detected by either of the six MLMs ranged 32–75, out of these, 11–44 QTNs were detected by at least two of the models concurrently (Additional File 2: Table S3). Out of the total 209 QTNs detected by six MLMs, thirty-nine were detected simultaneously in at least two environments with at least two models including *qHSW-13-26* & *qHSW-14-10* (Additional File 2: Table S2).

A total of 231 QTNs were identified by the two SLMs plus the six MLMs, out of these, seventeen were detected concurrently by the two categories of models (Additional File 2: Table S2; Additional File 5: Fig. S3B). *qHSW-2-1*, *qHSW-2-8*, *qHSW-2-10*, *qHSW-9-12*, *qHSW-13-8*, *qHSW-13-26*, *qHSW-14-10*, *qHSW-14-12* & *qHSW-17-4* were detected by more than of two the MLMs in at least three environments as well as by at least one of the SLMs in one environment (Additional File 2: Table S2). Pairwise comparison among the eight models showed vary range of QTNs (2–44) detection concurrently (Additional File 5: Fig. S3).

### Allele effect of QTNs on seed weight

**Fig. 5:**
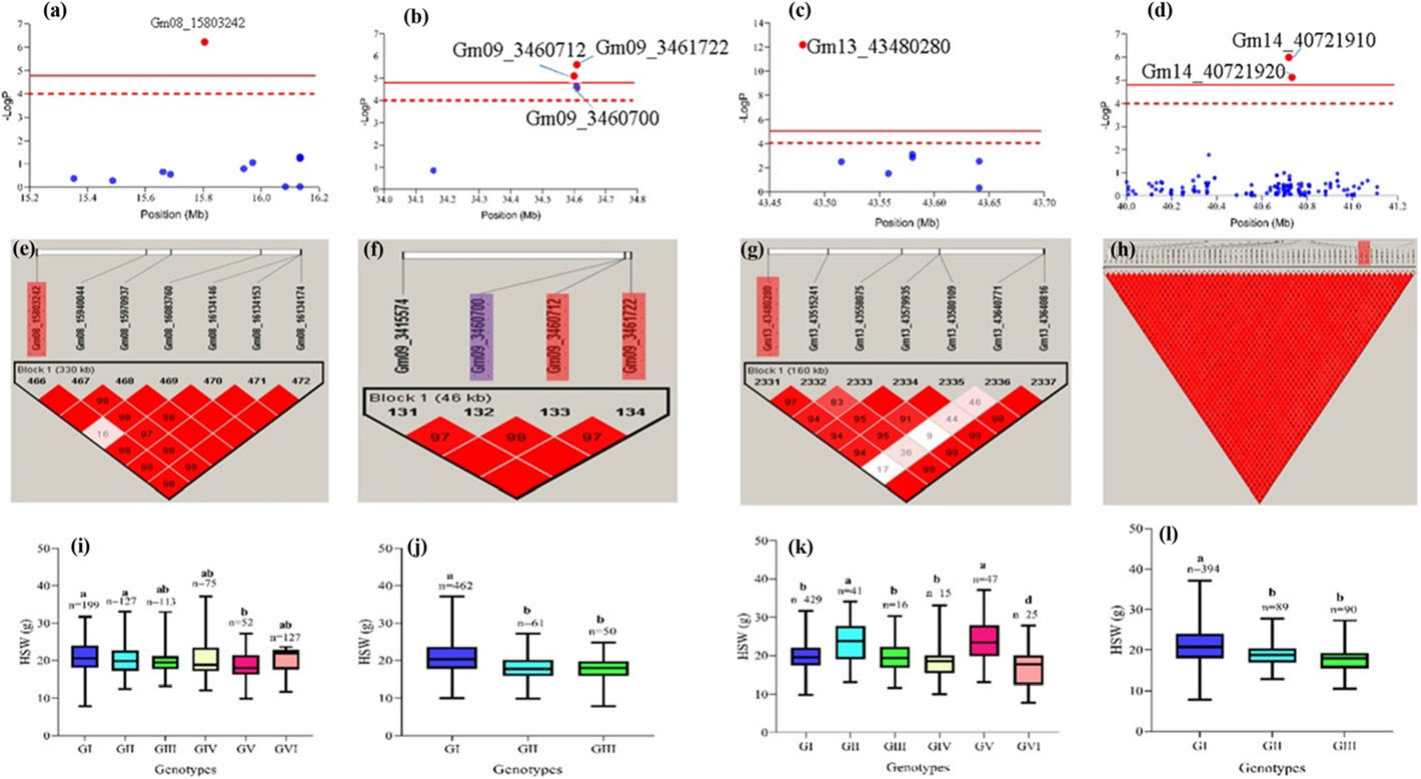
Manhattan plots, haplotype block analyses of selected QTNs and allele effect on seed weight (boxplot). *qHSW-8-8* are in **a**, **e** & **i**. *qHSW-9-4* are in **b**, **f** & **j.** *qHSW-13-26* are in **c**, **g** & **k**. *qHSW-14-10 & qHSW-14-11* are in **d**, **h** & **l**. The significant SNPs detected within each block are displayed in the Manhattan plots (**a**, **b** & **c**) and the dotted red line represents the reduced threshold (4) whereas the completed red line represents the Bonferroni correction threshold (4.79). The SNPs detected in each block are shaded red and purple for those that exceeded the 4.79 and 4, respectively. The boxplot for each of the blocks were obtained by the average seed weight across the 4 environments (E1, E2, E3 & E4) (I, J, K & L). The accessions were grouped and pairwise comparisons conducted by Duncan’s Multiple Range Test at *P < 0.05.* The boxes with a common alphabet indicate no significant difference in seed weight. Number of accessions (n) in each sub-class is represented on top of each box. GI-GVI represents a number of groupings of the 573 accessions in each block

Haplotype block analyses were conducted for *qHSW-8-8* (Gm08_15803242)*, qHSW-9-4* (Gm09_3461722*), qHSW-13-26* (Gm13_43480280) and two tightly linked SNPs, Gm14_40721910 (*qHSW-14-10*) & Gm14_40721920 (*qHSW-14-11*) in Haploview software with four gamete rule method (Fig. 5a-l). The distance within each block ranged 46–490 kb with range of 4–97 SNPs. The 573 accessions were grouped into 3–6 categories in each block with significant variation in seed weight.

### Candidate genes prediction and further analyses

Potential candidate genes were mined from 500 kb upstream and downstream of significant SNPs that were detected in at least two environments. SNPs within a block that were detected in at least one environment, only one was used to identify candidate genes. In all, thirty-six candidate genes were identified using the orthologs in Arabidopsis of which 14 and 22 were located upstream and downstream of the SNPs positions, respectively (Additional File 6: Table S4). Five SNPs had two potential candidate genes each, for instance, SNP at Gm08_15803242 (*qHSW-8-8*) had *Glyma08g20770* and *Glyma08g20780* at 38.65 and 27.86 kb downstream. These two genes code for ATP binding cassette (ABC) transporter protein with biological function of transport; transmembrane transport which have been demonstrated to play significant role in regulating seed size/weight with effect on seed yield [51]. These two genes together with *Glyma06g17520, Glyma06g44510, Glyma09g04840, Glyma11g27070, Glyma12g33280, Glyma13g18280* and *Glyma13g17890* are annotated to be involved in sugar/sucrose/ monosaccharide transport. The remaining 25 genes are involved in two or more biological processes such as cell proliferation, regulation of cell size, cell wall modification, flower/its part development, seed development, seed coat development and other biological processes which play key roles in regulating seed size/weight (Additional File 6: Table S4). From the RNA-Seq Atlas developed by Severin et al. [52] available on SoyBase database, it was discovered that all the predicted candidate genes are  highly expressed in seed-related tissues as well as seed developmental stages except *Glyma09g24020* (Additional File 6: Table S5).

The 1.5 kb upstream of each candidate gene was explored for seed-related regulatory elements. Three seed-related *cis*-elements (GCN4_motif, MSA-like and RY-element) were identified in the promoter regions of 13 of the predicted candidate genes (Additional File 6: Table S6). GCN4_motif [TGAGTCA] which is involved in endosperm expression was found in *Glyma01g38450, Glyma04g06760, Glyma13g17890, Glyma15g17040, Glyma15g39730* and *Glyma19g28070* [53]. MSA-like [(T/C)C(T/C)AACGG(T/C)(T/C)A] element which is involved in cell cycle regulation was found in *Glyma02g07240, Glyma09g04840* and *Glyma14g11930* [54]. RY-element [CATGCATG] involved in seed-specific regulation was identified in *Glyma03g06420, Glyma03g06600, Glyma06g17520* and *Glyma12g33280* [55]. Plant growth and development are regulated by circadian related genes especially flowering time. In addition, the circadian clock [CAAAGATATC] was detected in *Glyma01g38450, Glyma04g06760, Glyma06g44510, Glyma07g11550, Glyma08g20780* and *Glyma13g17890*. A study conducted by Hudson [56] demonstrated that the circadian clock controlled transcriptome of developing soybean seeds. Twenty of the predicted candidate genes had ABRE element involved in the abscisic acid responsiveness which have been reported to play a primary role in seed maturation [57].

**Fig. 6:**
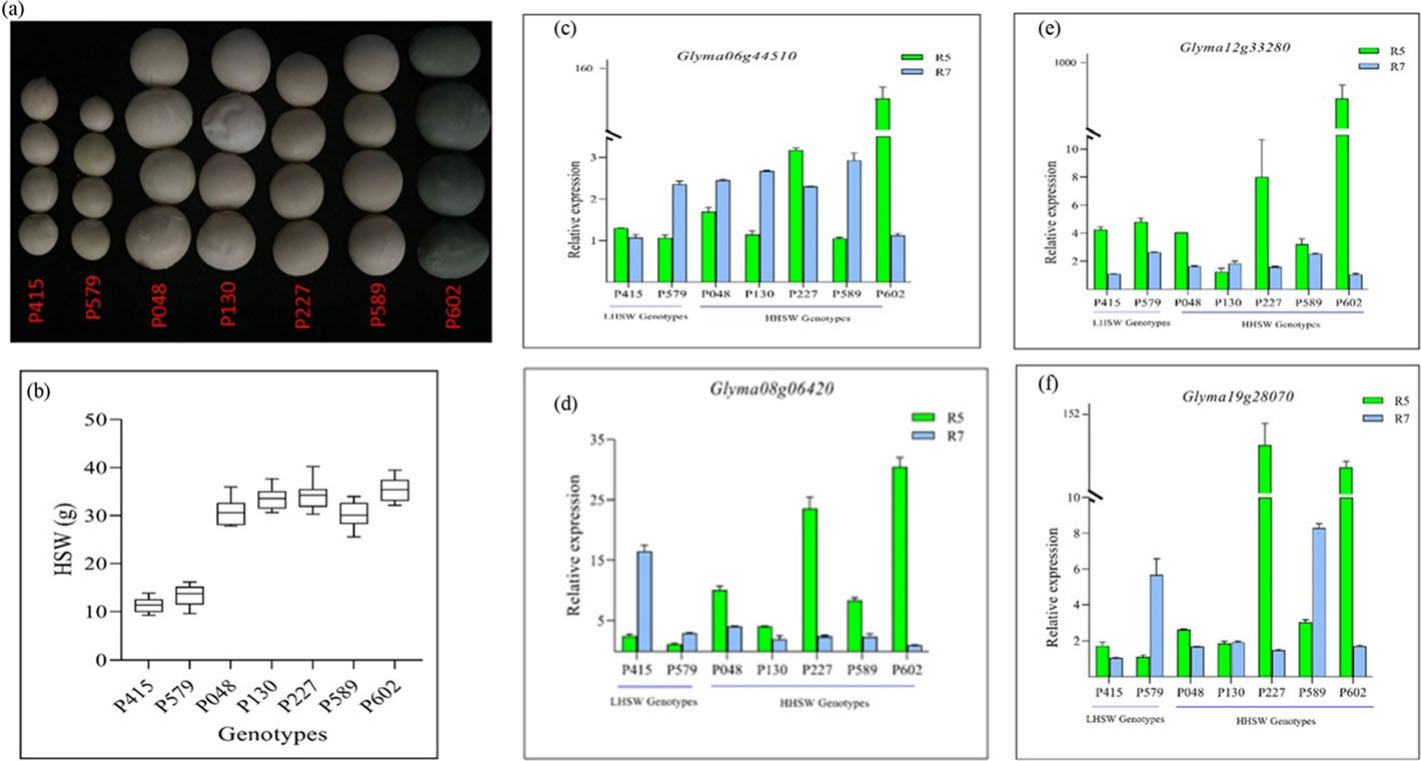
Seed weight among selected genotypes and relative expression of 4-hub genes by qRT-PCR. **a** & **b** Phenotypic characterization of selected genotypes for qRT-PCR. Relative expression of *Glyma06g44510* (**c**)*, Glyma08g06420* (**d**)*, Glyma12g33280* (**e**) and *Glyma19g28070* (**f**) by qRT-PCR with seed at R5 and R7 stages of seed development. (LHSW and HHSW represent Low HSW and High HSW, respectively. The error bars represent standard error of means)

To understand possible interaction among the 36 candidate genes whilst mining for other genes, the predicted candidate genes were subjected to SoyNet which has 40,182 soybean genes (73% of the coding genome) with two million functional network in soybean [58]. A dense interaction network among 213 genes distributed across the 20 chromosomes were found comprising 15 of the predicted genes in this study (Additional File 7: Fig. S4A&B). Four hub genes (*Glyma06g44510, Glyma08g06420, Glyma12g33280* and *Glyma19g28070*) in the network were among the predicted candidate genes. These hub genes were confirmed by qRT-PCR with seven extreme genotypes, thus, five high HSW genotypes (P048, P130, P227, P589 and P602) and two low HSW genotypes (P415 and P579) in seed sampled at R5 and R7 stages. With the exception of *Glyma12g33280,* the remaining three hub genes (*Glyma06g44510, Glyma08g06420* and *Glyma19g28070*) were relatively expressed higher in the high HSW genotypes at R5 stage compared with low HSW genotypes (Fig. 6). However, the expression were not consistent in the R7 stage.

## Discussion

### Phenotype variation and genetic basis of seed weight in YHSBLP

Identification of molecular markers associated with a trait of interest is one of the prerequisites of molecular breeding. Soybean seed weight is one of the most critical traits having direct effect on yield as a yield component, with much importance as a quality attribute and its influence on seed use [7–9]. The seed weight (HSW) ultimately determines the economic return on soybean production. However, this trait is a complex trait controlled by many polygenes with both major and minor effects, which is also significantly influenced by the environment and genotype by environment interaction. This makes screening on the basis of phenotype alone very difficult and inefficient. Hence, identifying QTL/QTNs for marker-assisted breeding for seed weight would be beneficial.

The HSW in soybean is a typical quantitative trait which is easily influenced by genotype, environment or by genotype × environment interaction (G × E) which are in consonance with several earlier studies [30, 46]. There was much variability in the YHSBLP (7.24–39.70 *g*) (Additional File 1: Table S1) which together with larger population size and high SNPs enhanced effectiveness and efficiency of QTN detection via SNP-trait association [49, 50, 59]. The HSW showed normal distribution in each environment (E1, E2, E3 and E4) (Fig. 1) coupled with high *h*^2^ which indicated that variation in HSW is controlled by multiple genetic loci with both major and minor effects (Table 1). This study utilized 573 recently developed breeding line population (YHSBLP) compared with most of the earlier studies that used wild accession, landraces and elite cultivars [24, 26, 27, 30, 46]. The high genetic variability shows the potentials of YHSBLP for genetic improvement aimed at seed weight [60].

### QTNs detected by the single and multi-locus models and their comparisons

Most of the earlier reported QTL/QTNs were performed with < 500 accessions and SNPs were < 60,000 [29, 30, 46–48]. The power of detection in GWAS is constrained by the population size, genetic diversity as well as genome coverage/number of SNPs, linkage disequilibrium, and statistical models [17, 49, 50]. Therefore, this study used two SLMs (MLM & CMLM) together with six MLMs (mrMLM, FASTmrMLM, FASTmrEMMA, pLARmEB, pKWmEB and ISIS EM-BLASSO) to identify genomic regions associated with seed weight. A total of 39 SNPs were significantly associated with HSW by the two single-locus models (MLM & CMLM) across ten out of the 20 chromosomes viz. Chr.01, Chr.02, Chr.04, Chr.08, Chr.09, Chr.11, Chr.13, Chr.14, Chr.15, Chr.16 and Chr.17 (Additional File 2: Table S2; Additional File 3: Fig. S1; Additional File 4: Fig. S1). Out of these, 24 were mutually detected by both MLM & CMLM whereas 6 and 9 SNPs were exclusively detected by CMLM and MLM, respectively (Additional File 5: Fig. S3A). On the other hand, two hundred and nine SNPs were associated with HSW via the six MLMs across the 20 chromosomes (Additional File 2: Table S2). Each of the six models detected varied number of SNPs: pLARmEB (82) > mrMLM (75) > pKWmEB (61) > FASTmrMLM (56) > ISIS EM-BLASSO (47) > FASTmrEMMA (32). This indicates varied detection of each model. Also, pairwise comparisons of the 6 models demonstrated that each of the models has the power to detect SNPs concurrently from each other, though no SNP was detected by all the six models simultaneously. For the example mrMLM & FASTmrMLM concurrently detected 44 SNPs followed by mrMLM & pKWmEB (22) and least by ISIS EM-BLASSO with FASTmrMLM (11) and FASTmrEMMA (11) (Additional File 2: Table S3). Multiple multi-site association analysis methods cannot only improve the reliability of QTNs detected, but also complement each other to detect more QTNs. In this study, two important QTNs: *qHSW-8-1* and *qHSW-19-4*/*qHSW-19-5* harbored 2 of the 4 hub genes identified were solely detected by only the MLMs. This also buttresses the usefulness of MLMs in GWAS.

In comparison of the two SLMs and six MLMs, 17 common SNPs were detected on 6 chromosomes (Chr.02, Chr.08, Chr.09, Chr.11, Chr.13 and Chr.14) (Additional File 5: Fig. S3B; Additional File 4: Fig. S2). Even though, the threshold of the significance in SLMs was adjusted to 4 instead of 4.79 (Bonferroni correction), the number of significant SNPs detected by SLMs were lower than those detected by MLMs, which confirm the robustness and power of detection of the later models. The combination of both SLMs and MLMs enhanced the detection of both major and minor QTNs. Several SNPs were detected in specific environment which are in consonance with ANOVA, indicating that seed weight of Chinese summer sowing soybean is also regulated by the environment. The combination of the two SLMs as well as the six MLMs complemented each other in identifying 231 QTNs which could have been lost in either of the models. However, the MLMs proved to be more robust and powerful in detecting more SNPs than the SLMs. Similar trend have been reported in soybean [34], cotton [35], maize [61] and flax [62] where more number of significant SNPs were detected by the MLMs comparing with SLMs. Therefore, the use of multi-locus models with their power of detection can facilitate genomic selection in breeding.

The stability of QTL/QTNs is essential for the use in a breeding program. Ninety-four of the QTNs identified in this study co-localized with several earlier reported QTL/QTNs could be exploited and integrated into breeding (Additional File 2: Table S2). Whereas 137 novel QTNs are being reported for the first time based on QTL/QTNs documented on SoyBase as some recently published reports need further verification. This could be attributed to the diverse background of our recently developed breeding lines pointing to their potential for breeding programs. Out of 137 novel loci, 22 were detected in at least two environments by at least two models (Additional File 2: Table S2). Also, allele effect on seed weight by haplotype block analyses could be utilized to conduct haplotype-based breeding scheme to develop genotypes with desirable seed weight whilst exploring the vast genetic base of YHSBLP.

### Candidate genes predicted and further analysis

Identification and utilization of candidate genes is one of the key objectives of GWAS. So far few genes have been validated and confirmed to regulate seed weight/size in soybean [63–65] compared with Arabidopsis and rice [66–71]. A mature seed consists of embryo, endosperm and seed coat derived from zygote, fertilized central cell and maternal integuments, respectively [67, 72]. Seed weight/size is also dependent on cell size and its proliferation, flower development, sucrose transport and other related activities which are regulated by several signaling pathways [66–68, 73–75]. Sucrose which is the major sugar composition accounts 97% in embryo during seed development process [76]. In our study, five of the thirty-six candidate genes are related with sucrose transport (Additional File 6: Table S4). For example, within the haplotype block Gm06_13796257–13926598 had a significant SNP at 13909376 bp (*qHSW-6-5*) with three sucrose transporter genes (*Glyma06g17520, Glyma06g17530* and *Glyma06g17540*) at 40.04 kb, 21.72 kb and 7.62 kb upstream of the SNP, respectively (Additional File 6: Table S4). These genes are 99.68, 99.62 and 99.61% similar to *AtSWEET12, AtSWEET13* and *AtSWEET10*, respectively. Again, another sucrose transporter gene *Glyma11g27070* which is ortholog to *AtSWEET15* was found 450.05 kb downstream of the SNP at 27075467 (Gm11_26861064-27075467). Less is known of the role of *GmSWEET* genes in seed development and its related traits in soybean [20, 77] compared to other crops [78, 79].

The functional network obtained from SoyNet revealed 4 hub-genes viz. *Glyma06g44510, Glyma08g06420, Glyma12g33280* and *Glyma19g28070* in our predicted candidate genes (Additional File 7: Fig. S4). Although, the functional relationship depicted in the co-expression network did not include all the 36 candidate genes predicted in this study, it gives better clues about 15 genes out of the predicted genes together with other genes which were not captured in this study. There is intensive literature on the interaction of ribosomal proteins and kinesin proteins in regulating embryo/seed size and radicle growth [80]. Such interaction was evident in the network constructed in this study. Some members of kinesin protein have been demonstrated to regulate embryo/seed size in rice [81, 82]. The network captured two genes (*Glyma17g17850* and *Glyma17g18360*) within the block Gm17_15178123-15400615 (SNP at 15346512). These two genes belong to Subtilase family protein and CYCLIN D3;2 which are involved in seed coat and other essential processes in seed weight regulation, respectively [73, 83]. Similarly, *Glyma10g38580* which is a K-box region and MADS-box transcription factor was captured in the network. MADS-box genes have been reported to be jack of all traits [84]. The network also covered the two genes from multidrug resistance-associated protein 6; ABC transporter transmembrane region; ABC transporter proteins which have recently demonstrated to enhance seed yield and quality in chickpea [51]. The functional co-expression network capturing some key genes which had some orthologs demonstrated to regulate seed development, implies that integration of co-expression can be one of the strategies to identify keys underlying major agronomic traits in crops.

*Cis*-acting regulatory elements (CAREs) are major switches for transcriptional regulation of a dynamic network of gene expression regulating different biological processes such as abiotic stress responses, hormone responses and developmental processes [85]. Further bioinformatics analysis showed that three of the four hub genes (*Glyma06g44510, Glyma12g33280, Glyma19g28070*) together with others possess seed-related *cis*-elements, thus, GCN4_motif, MSA-like, RY-element, circadian clock and ABRE element (Additional File 6: Table S6) [53–55, 86]. The predicted candidate genes especially the hub-genes will be validated in our future works to ascertain their actual roles in seed weight regulation via overexpression, CRISPR/Cas9 and other functional methods.

## Conclusion

The genetic architecture of YHSBLP planted in summer are regulated by varied QTNs unevenly distributed on the 20 chromosomes. Seventeen QTNs were detected concurrently representing 43.54 and 8.13% of the total QTNs by the two SLMs and six MLMs, respectively. Among the six MLMs, mrMLM and pLARmEB are most robust in detecting more QTNs. A number of SNPs including Gm08_15803242, Gm09_3461722, Gm13_43480280 and Gm14_40721910/ Gm14_40721920 detected by multiple models in at least two environments could further be validated and used for marker-assisted breeding (MAB). In all, thirty-six candidate genes that may regulate seed weight in soybean were identified. These will be useful in comparative genomics aimed at unraveling the molecular mechanism underlying seed development/weight in soybean. Four hub genes viz.*, Glyma06g44510, Glyma08g06420, Glyma12g33280* and *Glyma19g28070*, were identified by the integration of co-expression network. All the hub genes were found to have higher expression in the seeds of high HSW genotypes than low HSW at R5 stage except *Glyma12g33280*, therefore, they could be cloned to study their regulating role in seed development. The findings in this study would be valuable for breeding geared toward desirable seed weight via MAB and haplotype-based breeding scheme.

## Materials and methods

### Germplasm, field evaluation and phenotyping

The tested panel named as YHSBLP includes a total of 573 breeding lines adapted to Chinese Yangtze-Huai river region for both grain and vegetable soybean use. All lines were obtained from National Center for Soybean Improvement, Nanjing Agricultural University (NAU), Nanjing-China. This population was mainly derived from the core parents in breeding programs (Nannong 86–4, Nannong 88–48, Yuchu 4 and Nannongcaidou 5), other local and foreign elite cultivars. The hybrid method was used to select pods in F_2_-F_4_ generation of each combination, and the high-yield and good plant type were selected in F_5_-F_6_ generation. The lines selected for this study comprised high-yield and stable lines in F_8_-F_14_ generation.

Field evaluation of the population was conducted in Jiangpu (Latitude 33°03′ N; Longitude 118°63′ E), Experimental Station of NAU in summer 2013, 2014, 2017 and 2018 coded E1, E2, E3 and E4, respectively (Additional File 9: Table S8, or available in the National Center for Soybean Improvement website, http://ncsi.njau.edu.cn/info/1150/2069.htm). The lines were planted in randomized complete block design with 50 *cm* × 50 *cm* hill plots in 3 replications. All recommended agronomic and cultural practices were followed. A 100-seed weight (HSW) for each replication was measured with 2 technical repeats at 13% moisture content via electronic balance. The mean of each genotype from the 2 technical repeats were computed for each replication.

### Statistical analysis of 100-seed weight

Data collected were subjected to analysis of variance (ANOVA) in SAS (SAS Institute, 2010. SAS/STAT software version 9.2. SAS Institute Inc., Cary, NC) following statistical model:

$$ {y}_{ml o}=\mu +{G}_m+{E}_l+{GE}_{ml}+{R}_{o(l)}+{\upvarepsilon}_{ml o}, $$

where *y*_*mlo*_ stands for the individual observation of *mlo*^*th*^ experiment unit, *μ* is the total average HSW, *G*_*m*_ is the effect of the *m*^*th*^ genotype, *E*_*l*_ is the effect of the *l*^*th*^ environment, *GE*_*ml*_ is the interaction effect between the *m*^*th*^ genotype and the *l*^*th*^ environment, *R*_*o*(*l*)_ is the effect of the *o*^*th*^ block within the *l*^*th*^ environment, and ε_*mlo*_ is the residual error. All factors were considered as random.

Descriptive statistics such as mean, standard error of mean, kurtosis and skewness were computed in each environment with OriginPro 8 Statistical Software (Origin Corporation, Northampton, MA, USA) whereas variation in HSW among the genotypes was visualized using Violin plot with *ggplot2* package in R [87]. Broad-sense heritability (*h*^2^) were computed for the combined environment following ${h}^2={\sigma}_g^2/\left({\sigma}_g^2+{\sigma}_{ge}^2/n+{\sigma}_e^2/ nr\right)$ where ${\sigma}_g^2$ is the genotypic variance, ${\sigma}_{ge}^2$ is the genotype by environment interaction variance, ${\sigma}_e^2$ is the error variance, *n* is the number of environments, and *r* is the number of replications [88].

### Genotyping

The DNA sample of each accession was genotyped by the Restriction site-associated DNA sequencing (RAD-seq) technology to generate high throughput SNPs. Briefly, the genomic DNA of the 573 accessions was extracted from young leaves using the CTAB method [89]. All DNA fragments between 400 bp and 600 bp were obtained by TaqI digestion. The fragments were sequenced using an Illumina HiSeq 2000 instrument with a paired-end reads length of 90 bp(including 6 bp index) of read length [90]. All sequence reads were aligned against the reference Glyma.Wm82.a1.v1.1 [91] using SOAP2 software [92], and SNP calling was performed by RealSFS software [93]. The criteria filtering SNPs of the 573 accessions were as follows: a rate of missing and heterozygous allele calls ≤30%, minor allele frequency (MAF) ≥ 5%. The fastPHASE software [94] was used for genotyping the SNP imputation after the heterozygous alleles were turned into missing alleles, resulting in 61,166 high-quality SNP markers (Available on NCBI database: PRJNA648781, or available in the National Center for Soybean Improvement website, http://ncsi.njau.edu.cn/info/1149/2070.htm). The SNP density plot was constructed with *CMPlot* package in R [95].

### Genetic diversity, population structure and haplotype block

The filtered SNPs were further pruned using the –indep-pairwise command option of pLINK. The pruned SNPs were then used to estimate population structure via ADMIXTURE V1.3.0 software [96]. In the ADMIXTURE setting, the number of clusters (K) was set from 1 to 10 initially; then, each Q and the relevant *P*-value was estimated. The most likely number of subpopulations was determined using the method described in Evanno et al. [97]. Principal Component Analysis (PCA) was carried out in Trait Analysis by aSSociation, Evolution and Linkage (TASSEL) software, version 5.2 [98]. A pairwise Nei’s genetic distance matrix was calculated in TASSEL for Neighbor-joining tree construction.

### Association mapping and haplotype block analysis

Two SLMs of GWAS viz. MLM (Q + K) and compression MLM (CMLM)(PCA + K) were conducted in TASSEL 5.2 [98] and Genome wide Association Prediction Tool (GAPIT) environment in R [99], respectively, where Q matrix was obtained from population structure computed in ADMIXTURE V1.3.0 software [96], kinship matrix (K) was estimated in each software and 3 PCs were used for PCA in the CMLM. A threshold value (−*log*_10_(*P*) ≥ 4.00) was adopted to declare a significant association of SNPs with seed weight.

Six MLMs viz. mrMLM [100], FASTmrMLM [101], FASTEMMA [31], pLARmEB [102], pKWmEB [103] and ISIS EM-BLASSO [104] were computed in R with the package *mrMLM.GUI* (https://cran.r-project.org/web/packages/mrMLM.GUI/index.html). In these models, Q matrix was used to account for population structure whilst the kinship matrix (K) was computed in the mrMLM.GUI environment. A critical LOD value was set at 3. The SNPs detected by at least 2 models in least one environment was considered as relatively stable SPNs. QTL naming was done following the nomenclature of McCouch et al. [105], thus starting with ‘q’, followed by an abbreviation of the trait name (HSW, hundred seed weight) and the name of the chromosome, followed by the number of QTL detected on the same chromosome.

Haplotype block analysis of the relatively stable SNPs across the 2 of the single-locus plus at least 2 multi-locus models was conducted in Haploview software with the four-gamete rule method with default parameters in Haploview software version 4.2 [106, 107]. Duncan Range Multiple test (pairwise comparison) was used to assess variation in seed weight among accession groupings in each haplotype block at the significant level of *P ≤* 0.05.

### Candidate genes prediction and analysis

Potential candidate genes were retrieved within 500 kb of significant SNPs detected in at least 2 environments by either single-locus models or multi-locus models in the *G. max* William 82 reference gene models 1.0 in SoyBase [108]. The functional annotations of model genes downloaded from SoyBase which were screened manually. The predicted candidate genes were further compared with their orthologs in other legume crops to confirm their functions in relation to seed development using an Integrative Platform to study gene function and genome evolution in legumes(LegumePI) version 2 (http://plantgrn.noble.org/LegumeIP) [109] and Legume Information System (LIS) (https://legumeinfo.org/) [110].

The sequence of 1.5 kb upstream (before ATG) of each gene was obtained from Phytozome database (https://phytozome.jgi.doe.gov). The obtained sequences were then submitted to PlantCare database available on http://bioinformatics.psb.ugent.be/webtools/plantcare/html to identify *cis*-elements related to seed-related functions in the promoter region of each gene [111].

A functional network of protein-protein interaction among the predicted candidate genes and other related genes were obtained via SoyNet (https://www.inebio.org/soynet/serach.php) [58]. The functional network derived from SoyNet was then visualized in standalone version of Cytoscape software [112] and NetworkAnalyst 3 [113].

### Validation of hub genes by real time quantitative polymerase chain reaction (qRT-PCR)

Seven genotypes of YHSBLP with extreme differences in HSW (comprising 2 and 5 genotypes with low HSW and high HSW, respectively) were sampled at R5 and R7 stages of seed development [114] of 2019 summer season at Jiangpu Experimental station. Total RNA was isolated using Plant RNA Extract Kit (TIANGEN Co., Ltd. China) and complementary DNA (cDNA) synthesis obtained by using HiScript II QRT SuperMix for qPCR (+gDNA wiper) (Vazyme Biotech, Nanjing, China). The enzyme 2x ChamQ™ SYBR qPCR Master Mix Kit (Vazyme Biotech, Nanjing, China) was used following standard protocol and program in a Light Cycler 480 system (Roche, Roche Diagnostic, Basel, Switzerland). Three biological and three technical replicates were used. The *GmActin 11* (*Glyma18g52780*) was used as a housekeeping gene to normalize the relative expression level in R5 and R7 stages of the selected genotypes seed. The primers used for qRT-PCR are presented in Additional File 8: Table S7.

## Supplementary information

**Additional file 1 **: **Table S1.** Descriptive statistics of SW in YHSBLP evaluated in 4 environments.

**Additional file 2 **: **Table S2.** Quantitative trait nucleotides significantly associated with hundred seed weight identified by the single-locus models and multi-locus models. **Table S3.** Pairwise comparison of the 8 GWAS models.

**Additional file 3 **: **Fig. S1.** Manhattan plots (left) and QQ-plots (right) for GWAS of the 573 accessions for HSW in E1 (A), E2 (B), E3 (C) and E4 (D) using CMLM (PCA + K). The threshold of 4 was adopted with a blue line in the Manhattan plots. The X-axis represents chromosome number and Y-axis represents −*log*_10_(*P*). The X and Y axis in the QQ plots represent the expected and observed −*log*_10_(*P*), respectively. Red line in the QQ-plots with the shaded regions indicate a 95% confidence interval.

**Additional file 4 **: **Fig. S2.** Manhattan plots (left) and QQ-plots (right for GWAS of the 573 accessions for HSW in E1 (A), E2 (B), E3 (C) and E4 (D) using MLM (Q + K). The threshold of 4 was adopted with a blue line in the Manhattan plots. The X-axis represents chromosome number and Y-axis represents −*log*_10_(*P*). The X and Y axis in the QQ plots represent the expected and observed −*log*_10_(*P*), respectively. Red line in the QQ-plots with the shaded regions indicate a 95% confidence interval.

**Additional file 5 **: **Fig. S3.** Number of common significant SNPs detected between models. (A). A number of common SNPs detected by the two single-locus models (MLM-blue color & CMLM-brown color). (B). A number of common SNPs detected by the 2 single-locus models (MLM & CMLM-blue color) and six multi-locus models (mrMLM, FASTmrMLM, FASTEMMA, pLARmEB, pKWmEB and ISIS EM-BLASSO-brown color).

**Additional file 6 **: **Table S4.** Potential candidate genes underlying stable SNPs. **Table S5**. Candidate genes expression across the various tissues and seed developmental stages obtained from SoyBase. **Table S6.** Cis-acting regulating elements related to seed development obtained from PlantCARE.

**Additional file 7 **: **Fig. S4**. Functional gene network of candidate genes predicted in this study and other related genes obtained from SoyNet. (A). Dense-interaction network obtained from standlone version of Cytoscape software. (B). The 4-hub genes and other visualized in NetworkAnalyst version 3. The node colors represent between degrees of interaction: red, pink, purple and blue represent very high, high, moderate and low levels of interaction, respectively.

**Additional file 8 **: **Table S7.** Primers used for qRT-PCR.

**Additional file 9 **: **Table S8.** Phenotypic data (average) of 573 accessions used in this study.

## Data Availability

With the exception of SNP datasets, all data generated or analyzed during this study are included in this published article and its supplementary information files. The SNP dataset used in the current study are available in the Sequence Read Archive (SRA) at NCBI (SRA accession: PRJNA648781) repository and on the website of the National Center for Soybean Improvement, http://ncsi.njau.edu.cn/zygx.htm.

## Abbreviations

CAREs: *Cis*-acting regulatory elements
GWAS: Genome-wide association study
MLMs: Multi-locus models
QTL: Quantitative trait loci
QTNs: Quantitative trait nucleotides
RAD-seq: Restriction site-associated DNA sequencing
SLMs: Single-locus models
SNPs: Single nucleotide polymorphisms
