# Supplementary figures and images for: Identification of quantitative trait nucleotides and candidate genes for soybean seed weight by multiple models of genome-wide association study
Source: BMC Plant Biol. 2020 Sep 1;20:404. doi: 10.1186/s12870-020-02604-z (PMC7466808; doi:10.1186/s12870-020-02604-z)

**
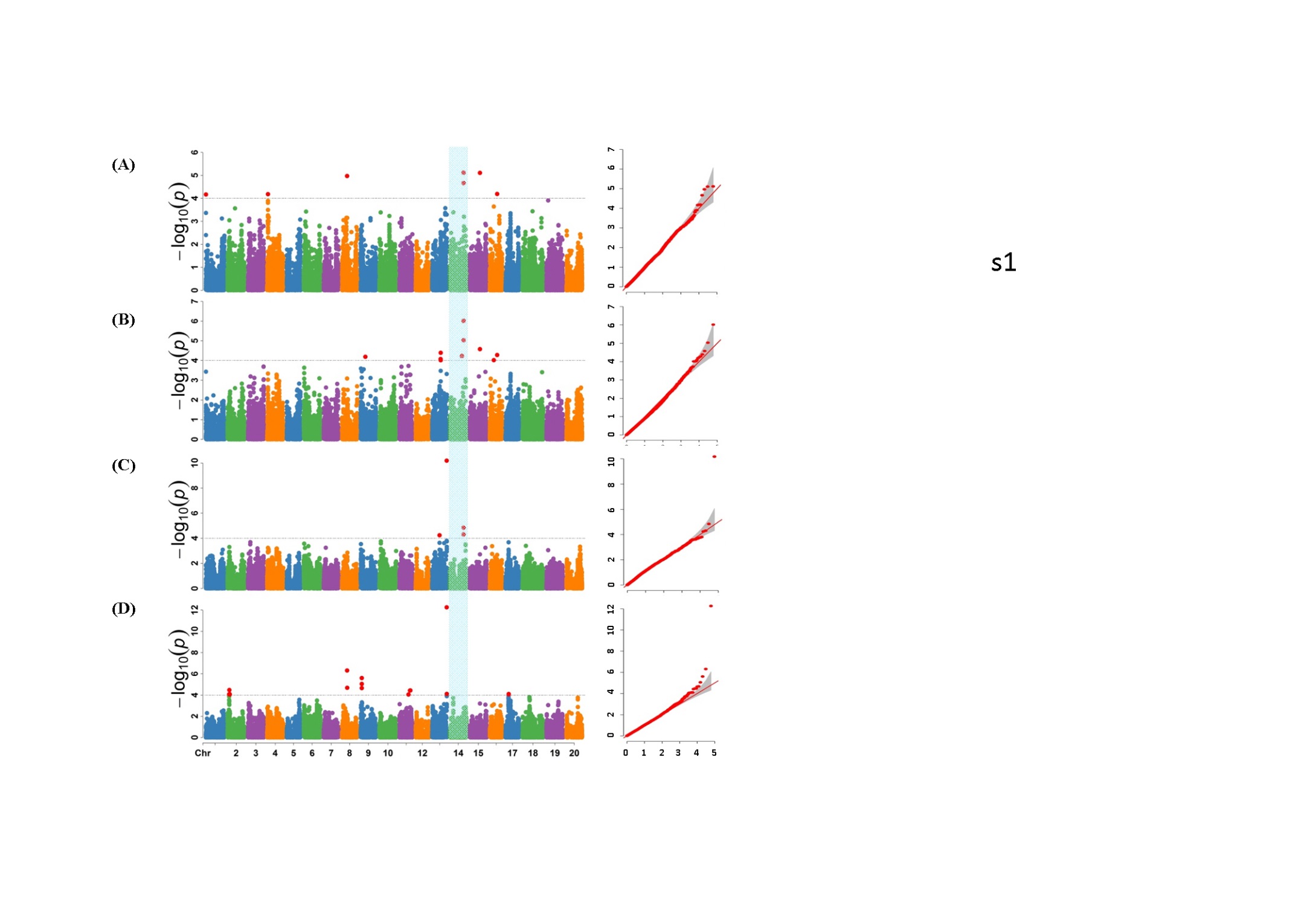
**

Supplement: Supplementary file 3 — Additional file 3 : Fig. S1. Manhattan plots (left) and QQ-plots (right) for GWAS of the 573 accessions for HSW in E1 (A), E2 (B), E3 (C) and E4 (D) using CMLM (PCA + K). The threshold of 4 was adopted with a blue line in the Manhattan plots. The X-axis represents chromosome number and Y-axis represents −log10(P). The X and Y axis in the QQ plots represent the expected and observed −log10(P), respectively. Red line in the QQ-plots with the shaded regions indicate a 95% confidence interval. [file 12870_2020_2604_MOESM3_ESM.docx]

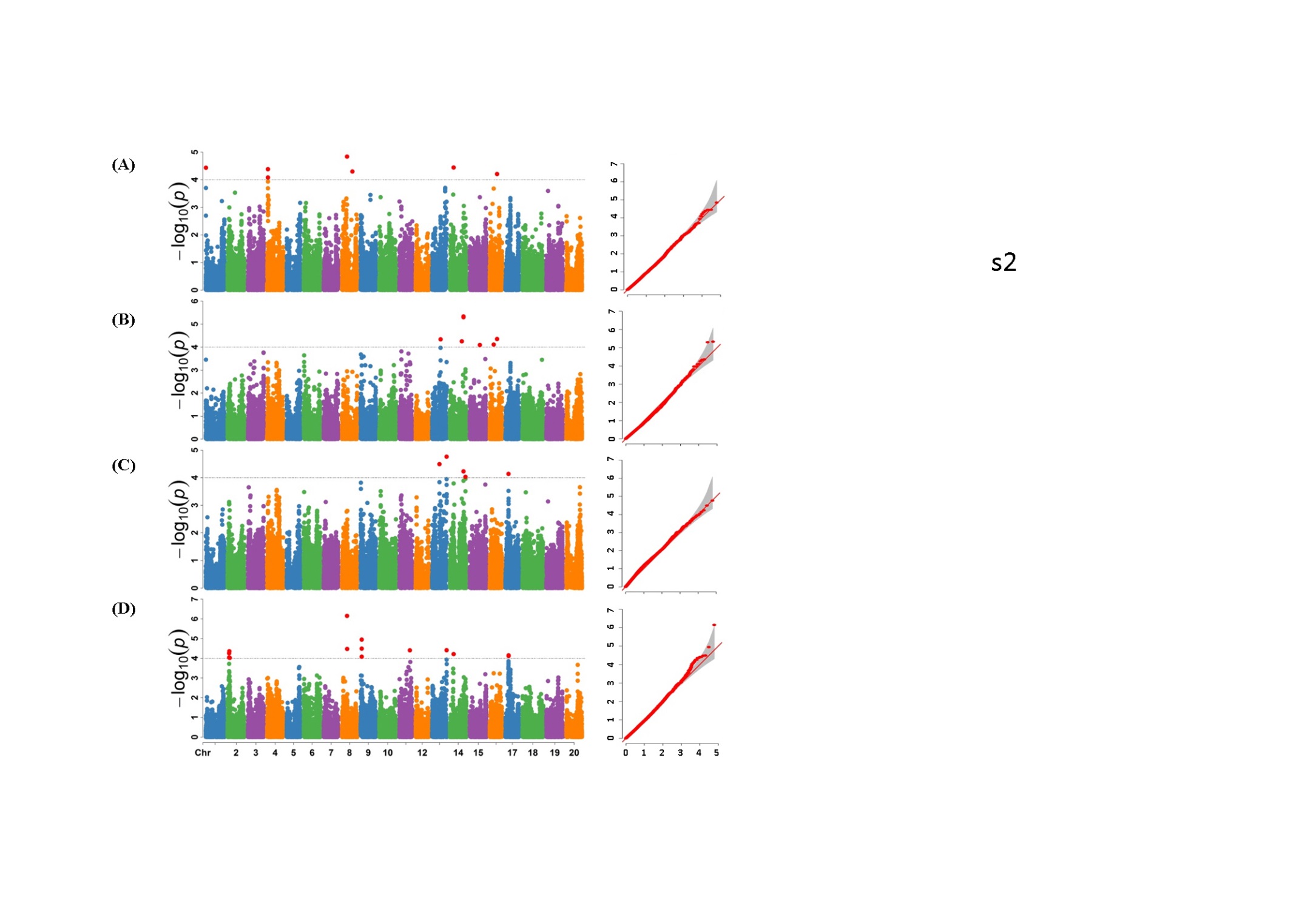

Supplement: Supplementary file 4 — Additional file 4 : Fig. S2. Manhattan plots (left) and QQ-plots (right for GWAS of the 573 accessions for HSW in E1 (A), E2 (B), E3 (C) and E4 (D) using MLM (Q + K). The threshold of 4 was adopted with a blue line in the Manhattan plots. The X-axis represents chromosome number and Y-axis represents −log10(P). The X and Y axis in the QQ plots represent the expected and observed −log10(P), respectively. Red line in the QQ-plots with the shaded regions indicate a 95% confidence interval. [file 12870_2020_2604_MOESM4_ESM.docx]

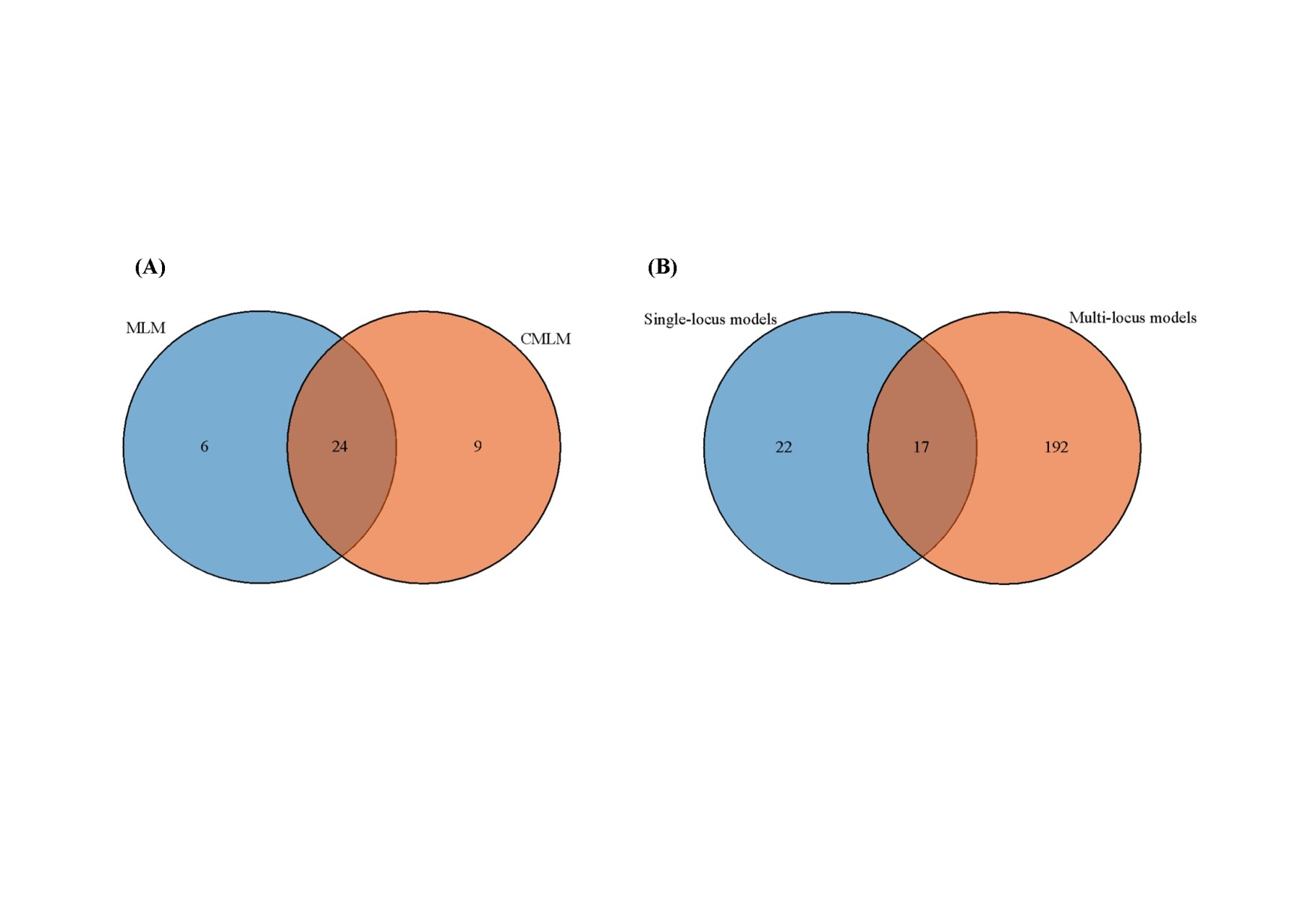

Supplement: Supplementary file 5 — Additional file 5 : Fig. S3. Number of common significant SNPs detected between models. (A). A number of common SNPs detected by the two single-locus models (MLM-blue color & CMLM-brown color). (B). A number of common SNPs detected by the 2 single-locus models (MLM & CMLM-blue color) and six multi-locus models (mrMLM, FASTmrMLM, FASTEMMA, pLARmEB, pKWmEB and ISIS EM-BLASSO-brown color). [file 12870_2020_2604_MOESM5_ESM.docx]

(A)


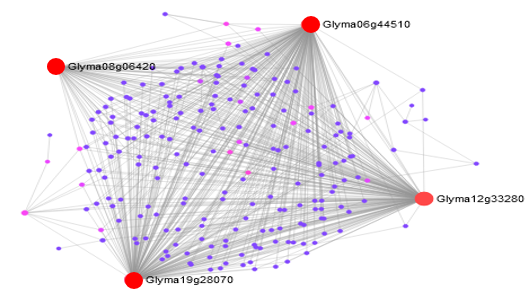


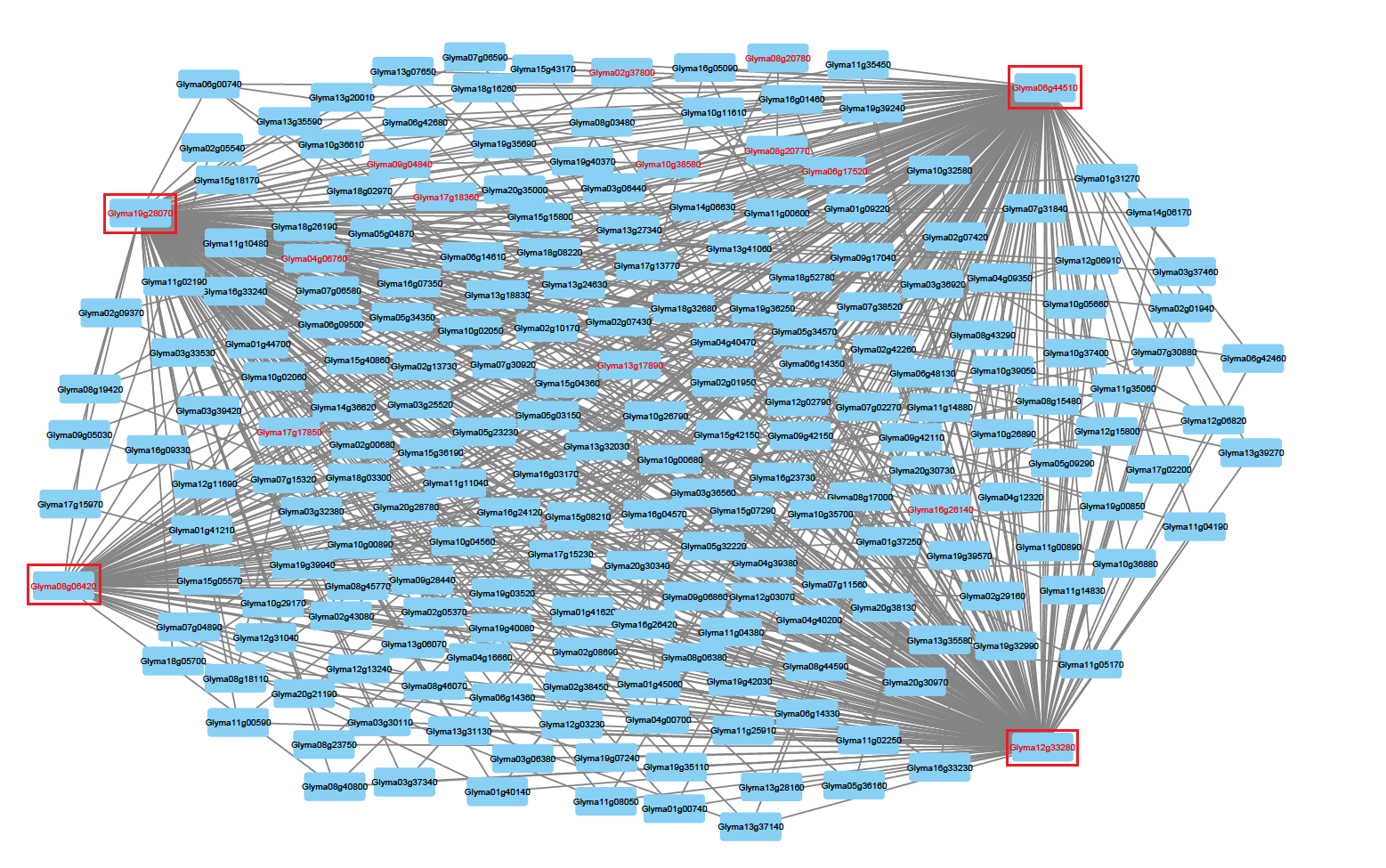


(B)

Supplement: Supplementary file 7 — Additional file 7 : Fig. S4. Functional gene network of candidate genes predicted in this study and other related genes obtained from SoyNet. (A). Dense-interaction network obtained from standlone version of Cytoscape software. (B). The 4-hub genes and other visualized in NetworkAnalyst version 3. The node colors represent between degrees of interaction: red, pink, purple and blue represent very high, high, moderate and low levels of interaction, respectively. [file 12870_2020_2604_MOESM7_ESM.docx]
